# MRE11-RAD50-NBS1 complex alterations and DNA damage response: implications for cancer treatment

**DOI:** 10.1186/s12943-019-1100-5

**Published:** 2019-11-26

**Authors:** Lei Bian, Yiling Meng, Meichao Zhang, Dong Li

**Affiliations:** Department of Radiation Oncology, Shanghai Ninth People’s Hospital, Shanghai Jiaotong University School of Medicine, Shanghai, China

**Keywords:** MRN complex, DNA damage response, tumorigenesis, chemotherapy, radiotherapy

## Abstract

Genome instability is a hallmark of cancer cells and can be accelerated by defects in cellular responses to DNA damage. This feature of malignant cells opens new avenues for tumor targeted therapy. MRE11-RAD50-NBS1 complex plays a crucial role in sensing and repair of DNA damage. Through interacting with other important players of DNA damage response, MRE11-RAD50-NBS1 complex is engaged in various DNA damage repair pathways. Mutations in any member of this complex may lead to hypersensitivity to genotoxic agents and predisposition to malignancy. It is assumed that the defects in the complex may contribute to tumorigenesis and that treatments targeting the defect may be beneficial to cancer patients. Here, we summarized the recent research findings of the role of MRE11-RAD50-NBS1 complex in tumorigenesis, cancer treatment and discussed the potential approaches of targeting this complex to treat cancer.

## Background

From endogenous processes that induce DNA damage, such as reactive oxygen species and replication fork collapse, to exogenous genotoxic agents that attack DNA and produce various DNA lesions, such as chemical agents, ultra violet and ionizing radiations, human genome stability is challenged constantly in daily life [1]. These lesions need to be repaired to prevent loss of genome information or faulty transmission to the next generation. If not repaired properly, they may give rise to mutations or chromosomal damage and can ultimately cause abnormalities, including oncogenic transformation and tumor progression [2]. For the maintenance of genomic stability, organisms have developed various mechanisms to cope with these damages. DNA damage response (DDR) refers to a plethora of various signal pathways and at least 450 related proteins [3, 4]. Activation of DDR halts cell cycle progression, leaving time for repair or bypass of damage. It also impacts on downstream cell fate decisions, inducing senescence, apoptosis or elimination by immune system [5–7]. On the other hand, genome instability is a hallmark of tumor cells [8] and is associated with a proneness to accumulate DNA damage in malignant cells. Meanwhile, a main difference between normal cells and cancer cells is that most cancer cells already harbor one or more DDR defects during their transformation, which confers an increased dependency on the remaining mechanism [4]. This hallmark of cancer cells outlines an opportunity to treat malignancies by targeting remaining DDR that they depend on [9, 10].

**Fig. 1:**
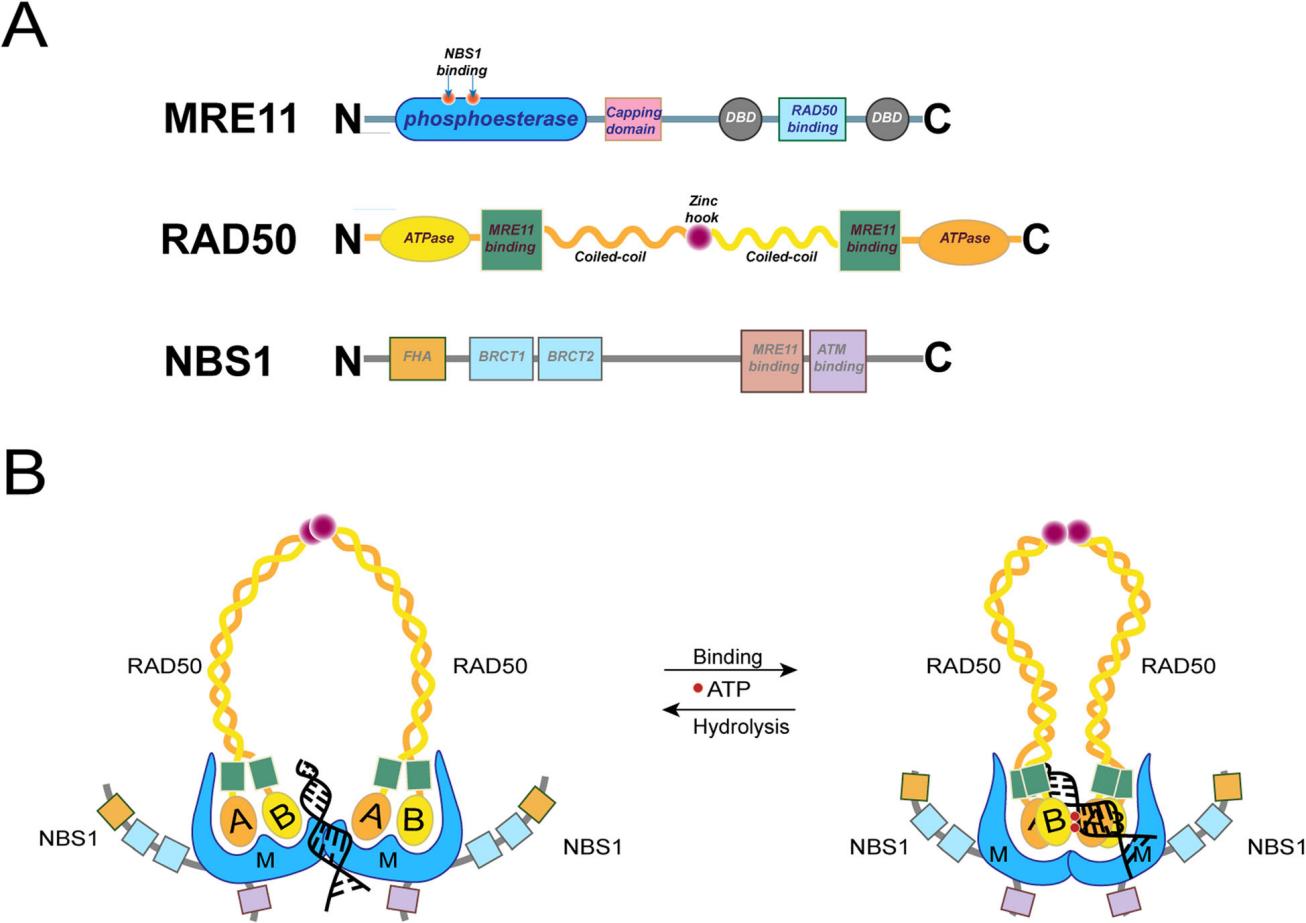
Structure and conformation of MRN complex. **a** Key domains of MRN complex subunits. Phosphoesterase domain of MRE11 has both single strand DNA endonuclease and double strand DNA exonuclease activity. Antiparallel coiled-coil domain of RAD50 extends the protein. Zinc hook domain of RAD50 facilitates the formation of dimers as depicted in (**b**). **b** MRN complex goes through conformational changes when ABC-ATPase domains (A/B Walker motifs) binds to ATP and forms a head-to-tail dimer. This compact, rigid, and closed conformation blocks access to MRE11 active sites. Upon hydrolysis and removing of ATP, MRN complex switches to an open form, exposing the active sites of MRE11. (FHA: fork-head associated domain; BRCT: breast cancer-associated 1 C domain)

The MRE11-RAD50-NBS1 (MRN) complex has a crucial role in DDR. It is one of the first sensors and responders to DNA damage and orchestrates DDR response in double-strand break (DSB), replication fork (RF) collapse, dysfunction of telomeres and viral invasion [11]. At the molecular level, MRN is composed of two meiotic recombination 11 homolog 1 (MRE11) subunits, two ATP-binding cassette (ABC)-ATPase (RAD50) units, and two phosphopeptide-binding Nijmegen breakage syndrome protein 1 (NBS1) subunits (Fig. 1a). Insights from structural analysis of MRN complex demonstrate that the assembly of hetero-hexamers possesses multiple biochemical functions including: ATP hydrolysis of RAD50 [12, 13], DNA binding by multiple units, bridging the DNA molecules together along with exonuclease and endonuclease activities.

In addition to these activities, MRN triggers cell cycle checkpoint response by interacting with ataxia-telangiectasia mutated (ATM) and ATM-and-Rad3-related (ATR) proteins [14], which are essential components of DDR [15, 16]. ATM initiates a variety of cellular signaling pathways in response to DNA damage, including cell cycle control, transcription and DNA repair [17]. MRN complex plays an important role in modulating ATM activity. In MRN deficient cells, ATM activation is defective [18]. Subsequent studies found that NBS1 plays an important role ATM activation [19].

Based on DDR deficiency in tumorigenesis and tumor development and the intriguing potential function of MRN complex in DDR for cancer treatment, we sought, in this review, to focus on the structure and biofunctions of MRN complex and its roles in tumor development, treatment efficiency and the potential as a target for anti-cancer therapy.

### Structure and function of MRN complex

The MRN complex controls biological outcomes of DNA damage by acting as flexible scaffold, combined sensor, signaling and effector complex via its dynamic states. It impacts three critical functions in processing of DNA stress: DNA binding and processing, DNA tethering to bridge DNA over short and long distance, and activation of DDR response and checkpoint signaling pathways. Its structure architecture underlies these diverse roles [20].

#### Structure of MRN complex

MRN structure can be separated into “head”, “coil”, “hook” and attached adapter regions.

The head region consists of two RAD50 ABC-ATPase domains and two MRE11 nucleases bound to the base of the RAD50 coiled-coils (Fig. 1b). It functions as the DNA binding and processing core of MRN complex. MRE11 contains an N-terminal nuclease motif followed by a cap domain that restricts access to the nuclease site, a RAD50 binding motif and C terminal DNA binding domains (DBDs) (Fig. 1a) [21, 22]. The nuclease and DBDs enable the 3’-5’ dsDNA exonuclease activities and 5’-3’ endonuclease activities which resects DNA ends to generate 3’ single-stranded DNA overhangs for further repair [23, 24].

The globular RAD50 ATPase domains are formed by two halves encoded at either end of the primary sequence. These two halves sequence which come together to form the globular “head” from the collapse of the inserted intramolecular antiparallel coiled-coil sequence (Fig. 1a) [13, 25]. This coiled-coil region forms a long (~500 Å) extension from the head of the MRN complex with a central CxxC motif forming a Zn-hook domain at the apex of the coiled-coil. This Zn-hook mediates Zn-dependent RAD50 subunit interaction and formation of flexible bridge of various distances (up to 1200 Å) [13, 26]. RAD50 conformation at the dimer interface is controlled by the binding of ATP molecules [13].

The NBS1 subunit is specific to eukaryotes. In eukaryotes, NBS1 forms the flexible adaptor domain of MRN and acts as a regulator and protein recruitment module. It consists of one fork-head associated (FHA) and two breast cancer-associated 1 C (BRCT) domains which form the phosphorylated protein binding core, a MRE11 binding motif and an ATM binding motif (Fig. 1a). NBS1 renders MRN complex its signaling role through binding to ATM or ATR in the context of DSB or replication fork stalling, respectively [27, 28].

**Fig. 2:**
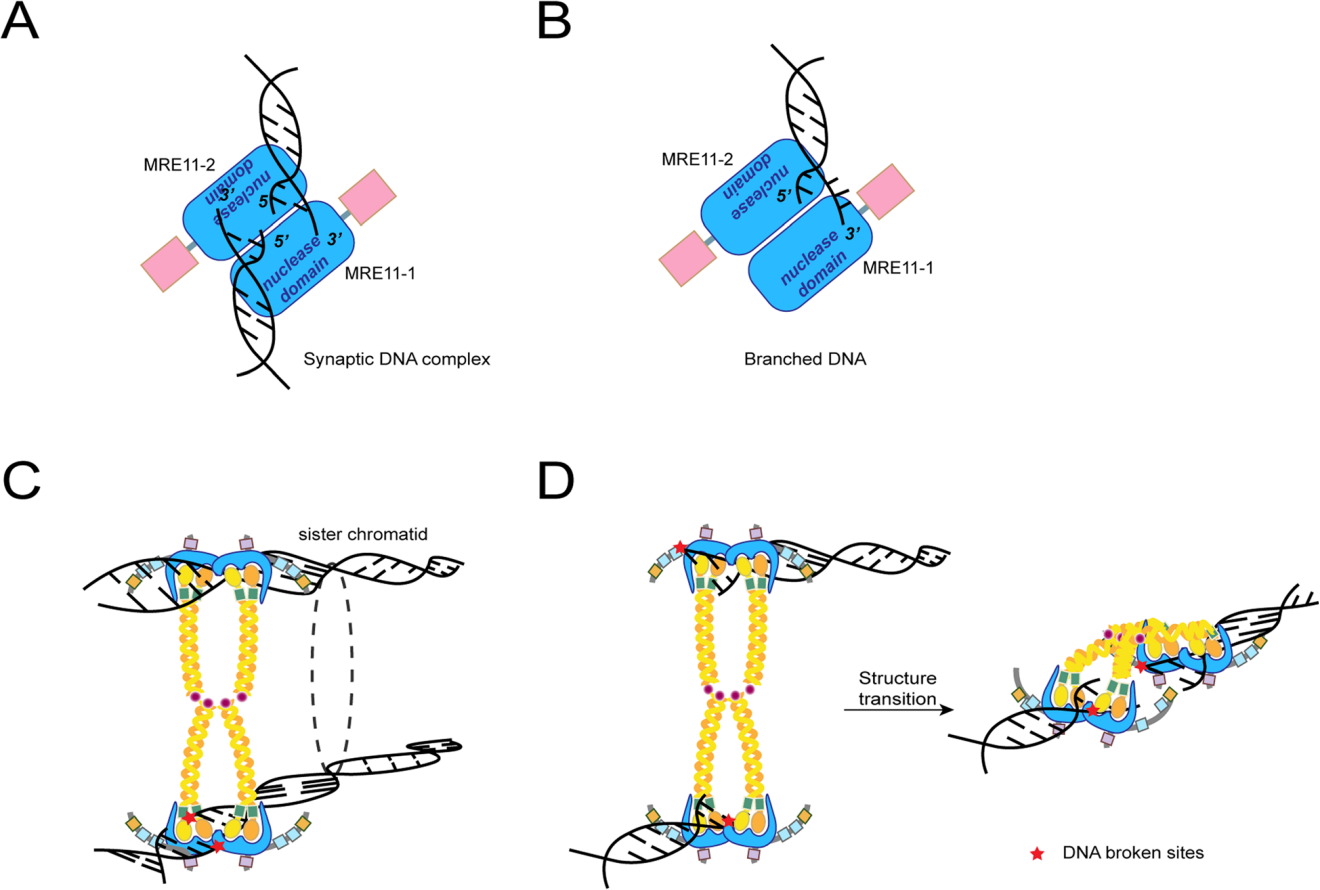
DNA binding and processing function of MRN complex. **a**-**b** Vertical view of MRE11 complex holding synaptic DNA complex (**a**) and branched DNA (**b**). Two MRE11 subunits are oriented symmetrically and bind the synaptic DNA complex which is normally seen in HR (**a**). In collapsed replication fork, branched DNA is hold in MRE11 complex asymmetrically in one half of the MRE11 dimeric cleft (**b**). **c-d** Models for MRN complex in processing DNA ends. For initiation of HR, MRN complex binds sister chromatid with broken DNA through a tail-to-tail link with another MRN complex and prepares for the further procedure of HR (**c**). In NHEJ, two MRN complexes bind the two ends of broken DNA separately. Later, through structure transition, MRN complexes tether and align broken ends for subsequent repair steps (**d**)

MRN complex goes through conformational changes when ATPase domain of RAD50 binds to ATP. It closes its arms and forms a compact, rigid, and closed conformation which can accommodate dsDNA but with the access to MRE11 nuclease sites blocked. In the absence of hydrolysis state of ATP, the RAD50 ATPase subunits are flexible and relatively open, and this renders DNA accessible to the MRE11 nuclease active sites (Fig. 2b) [29].

The diverse DNA scaffolding functions of MRN complex is dictated by MRE11 [21]. In the synaptic end complex, two broken DNA ends are housed in near parallel yet opposed trajectory. This linear bridging provides a molecular mechanism to tether DNA ends within short distances (Fig. 2a) [13, 30]. In the branched complex mainly found in RF stalling, the branched DNA binds asymmetrically across the dimer [21] (Fig. 2b).

#### The function of MRN complex

The MRN complex is the core conductor for the initial and sustained response to DSB, stalled RF, dysfunctional telomeres and immune responses to viral DNA infections [11].

##### The MRN complex and DSB repair

The role of MRN in DSB repair is extensively studied. It functions in both DSB sensing and repair. In sensing stage, MRN complex is recruited to broken sites by γ-H2AX and RAD17 [31], both of which interact with NBS1 [32]. At DSB sites, MRN activates ATM through the interaction between ATM and NBS1. Then ATM activates and regulates a DSB-cascade [16]. MRN also orchestrates the pathway choice in DSB repair. There are two major pathways through which cells repair DSBs: the template-dependent error-free homologous recombination (HR) and error-prone canonical nonhomologous end joining (c-NHEJ). It is suggested that MRN complex licenses HR pathway choice through an MRE11 endonuclease cut, which generates 3’ ssDNA overhangs that inhibits NHEJ. This is followed by MRE11 exonuclease and EXO1/BLM bidirectional resection toward and away from the DNA end, which commits to HR [24]. Nonetheless, the biochemical endo- and exonuclease activities of MRN also act on DNA ends with protein adducts [33]. It removes covalently linked protein complexes such as topoisomerase complexes from genomic DNA [34] and might facilitate the repair by NHEJ [35]. In HR and NHEJ, DNA tethering and bridging function is mainly facilitated by the Zn-hook in RAD50 [13, 36]. In HR, MRN complex is tail-to-tail linked (Fig. 2c). This structure facilitates the bridge of sister chromatid and formation of stabilization of displacement loop, a common intermediate in recombination, break-induced replication and telomere maintenance [13]. In NHEJ, the head domains of two MRN complexes bind separate DNA ends, aligning and tethering them after a structure transition (Fig. 2d).

##### The MRN complex and replication stress

Collapse of RF can be induced by different causes and activate the replication stress response, which is primarily mediated and solved by ATR [37]. MRN complex entitles a dual role in response to replication stress. It binds DNA structures or breaks encountered in stalled or collapsed RF and promotes the restart of DNA replication and activates ATR [38, 39]. Also, MRN-mediated resection of nascent DNA likely frees the replisome from the collapsed RF [40] and generates 3’ overhangs for initiation of HR, which in turns restores the replication under stress but possibly contributes to genomic instability [41]. On the other hand, unregulated resection can be detrimental and lead to fork degradation mediated by MRE11 exonuclease activity [42]. MRE11 nuclease activity could be inhibited by BRCA2 while the absence of BRCA2 may lead to chemoresistance [43, 44]. Nonetheless, limiting the access of MRE11 to ssDNA at stalled RF may also contribute to the development of chemoresistance [45]. This duality highlights the complexity by which tumor cells evade chemotherapeutics and develop resistance. However, it also provides a potential mechanism that synthetic lethality can be induced.

##### The MRN complex and dysfunctional telomeres

In humans, the MRN complex also plays dual roles at telomeres. It assists the shelterin complex to avoid DSB repair at functional telomeres, thus suppressing tumor development [46, 47]. Otherwise, it causes genomic instability by promoting NHEJ pathway at dysfunctional telomeres. NBS1 and MRE11 may participate in these processes [11]. Also, the MRN complex regulates telomere length, most likely by recruiting telomerase [48]. A better understanding of the biochemical role of MRN in dysfunctional telomeres needs future experimentation.

##### The MRN complex and viral infections

Upon viral infection, viral DNA in the cytoplasm is recognized and bound by MRN complex, and the complex signals local ATM activation that specially inhibits viral replication without escalating the response to a global one [49]. MRN binding to viral DNA in the cytoplasm also leads to production of type I interferons [50]. However, some viral encoded proteins are capable of inactivating MRN complex through protein relocalization and degradation [51, 52]. The MRN complex may also have impact on immune system development by controlling class switch recombination, although the results from different reports are controversial [53]. Based on the interplay between viral infection, DNA repair and immune responses, oncolytic virus-based therapies targeting the MRN complex are implemented in the treatment of cancers as discussed below.

### MRN complex alterations and tumorigenesis

MRN complex is involved in different responses to cellular damages and plays a crucial role in orchestrating DDR. Meanwhile, each component of this complex owns important biological roles. Disruption of either component of the complex in mice results in embryonic lethality [54–56]. In humans, hypomorphic mutations in any of these three genes can cause syndromes associated with genome instability [57]. For instances, mutations in MRE11 gene lead to ataxia-telangiectasia-like disorder (ATLD), which is quite rare with only a few cases reported [58–61]. Mutations in NBS1 gene cause Nijmegen break syndrome (NBS) [62, 63], which is an autosomal recessive genetic disorder. Both ATLD and NBS are characterized by immunodeficiency, genomic instability, hypersensitivity to radiation and predisposition to cancer. RAD50 deficiency has been reported in a case of NBS disorder [64]. Based on the fact that all the three components of MRN are implicated in cancer-prone disorders, we postulated that alterations in MRN expression or function may be implicated in malignant transformation.

While mutations in BRCA1/2 are the most common alterations implicated in hereditary cancer syndromes [65, 66], mutations in other genes are identified increasingly in cancer patients with the development of multigene panel testing [67].

MRE11, RAD50, and NBS1 genes were identified as genes with moderate penetrance for breast cancer [68]. In a mice model of sporadic onco-gene-driven breast tumorigenesis, impairment of the functions of Mre11 complex promotes the progression of mammary hyperplasia into invasive, metastatic breast cancer while intact Mre11–mediated DDR restrains progression of mammary hyperplasia [69]. Recently, a pooled analysis from a large case-control study reveals that carriers of MRN variants present increased breast cancer risk [odds ratio (OR)=2.9] [70]. In this study, missense substitutions in MRE11, RAD50 and NBS1 key functional domains have been observed. Similar results have also been reported by various groups [71–73]. However, controversial findings exist. In a multigene panel-based clinical testing for pathogenic variants in inherited cancer genes, the genes of MRN complex do not confer any appreciable risks of breast cancer [74]. Associations between mutations of MRN complex genes and cancer susceptibility have also been observed in ovarian cancer [75, 76] and glioma [77].

Alterations of single gene of the MRN complex have also been reported in cancer development. MRE11 negativity is significantly higher in sporadic gastric cancer than in early-onset or familial gastric cancer [78]. In a chemical reagent-induced mouse malignancy model, the expression level of Mre11 in tumor tissue is significant lower than that in non-tumor tissues, suggesting that chemical reagents might induce changes in DDR proteins and that the alterations in DDR gene products are important for tumor development and growth [79]. It is also reported that alterations of MRE11 are frequent in neuroblastoma [80].

RAD50 is reportedly altered in acute myeloid leukemia [81], endometrial carcinoma [82], Burkitt lymphoma [83] and so forth. The homozygous mutation in the Zn-hook domain of Rad50 is lethal in mice, while the heterozygous mutation leads to liver tumorigenesis, suggesting that Rad50 hook domain strongly influences tumorigenesis [84].

NBS1 has emerged as a prostate cancer-susceptibility gene, with its variant being more prevalent in patients with familial history rather than sporadic prostate cancer [85]. The relation between NBS1 variant and increased cancer susceptibility is also observed in lung cancer [86], liver cancer and intrahepatic cholangiocarcinoma (IHC) [87].

For better clarification, whether these variants can lead to altered MRN function deserves further studies. It has been reported that alterations in the BRCT domain of NBS1 compromises its interaction with BRCA1 and genome surveillance ability, which is associated with susceptibility to other tumors [88]. Previous reports also showed that the presence of variant allele might lead to a lower MRN complex activity, leading to an impaired cellular ability to detect and repair the DNA damage [89, 90]. Further exploration of the relation between MRN variants and its function may help to depict the link between MRN alterations and tumorigenesis.

With respect to the link between MRN alterations and tumorigenesis, there are still several questions that need to be addressed.

First, the frequency of gene variations of MRN complex is about 1% or less in cancer patients [91–93], suggesting that cooperation with other genes is needed when these genes are considered as testing panel.

Second, at present, the role of MRN complex in tumorigenesis still remains controversial, due to studies that led to opposite conclusions related to its tumor suppressive role [94–96]. This may be caused by the lack of standards for evaluation of MRN complex and reliable way to treat MRN complex as a whole rather than measure individual component separately. There is a strong correlation among MRE11, RAD50 and NBS1in complex formation; modulation of single component of the complex can impact the entire complex [97].

Third, most studies have focused on the relationship between inherited gene mutations and cancer susceptibility risk [68, 85, 88]. On one side, they provide detailed information about genetic variations and predisposition to malignancy in populations with cancer family history, which may facilitate cancer prophylaxis. On the other side, more studies are needed to unveil the role of MRN in sporadic cancer to better understand this complex and explore its potential for treatment.

Last of all, MRN complex is postulated as a potential tumor suppressor because of its indispensable role in DDR and maintenance of genomic stability. This role may also involve counteracting oncogene-induced replication stress in cancer cells [98]. In normal cells, MRN complex suppresses genome instability and prevents accumulation of DNA damages. However, the intact function of MRN complex is far beyond its tumor suppressor function. It may also contribute to cell survival in tumor cells [98]. Thus, when the role of MRN complex in tumorigenesis is interpreted, its function in cancer cells must be taken into consideration.

Recent publications related to the role of MRN complex in tumorigenesis are listed in Table 1.

**Table 1:** Role of MRN complex in tumorigenesis

Cancer type	Molecules	Results	Year	Ref
B cell lymphoma	MRE11	x MRE11 promotes tumorigenesis by facilitating resistance to replication stress.	2017	[98]
Breast Cancer	MRN	✓ MRE11, RAD50 and NBS1 were genes with moderate penetrance.	2016	[68]
	MRN	✓Mutations in MRN contribute to increased cancer risk.	2013	[70]
	MRN	x MRN complex genes did not confer any appreciable risks.	2017	[74]
	MRE11	✓ Defect in MRE11 function promotes progression of mammary hyperplasia into invasive, metastatic cancer.	2013	[69]
	MRE11	✓ MRE11 variant was associated with increased risk of breast cancer.	2018	[73]
	MRE11, RAD50	✓ MRE11 and RAD50 were moderate-susceptibility genes for breast cancer.	2015	[92]
	MRE11	x Variants of MRE11 gene did not constitute a risk factor.	2018	[96]
	NBS1	✓ NBS1 variant 923T>C might be a risk factor for breast cancer development.	2016	[72]
Burkitt lymphoma	RAD50	✓ RAD50 mutations were identified in Burkitt lymphoma.	2017	[83]
Endometrial carcinoma	RAD50	✓ RAD50 mutation was frequently detected.	2017	[82]
Gastric cancer	MRE11	✓ Low expression of MRE11 was associated with gastric cancer.	2019	[78]
Glioma	MRE11, NBS1	✓ NBS1 and MRE11 variants were associated with increased risk	2015	[77]
HBOC	MRN	✓ MRN mutations were detected in HBOC patients.	2014	[93]
	RAD50	✓ Mutations in RAD50 was implicated in cancer predisposition.	2017	[71]
Hepatic carcinoma	MRE11	✓ Expression of MRE11 was lower in chemical reagent induced hepatic carcinoma.	2015	[79]
Hepatic carcinoma	RAD50	✓ Mutations in RAD50 lead to liver tumorigenesis.	2014	[84]
IHC	NBS1	✓ NBS1 variants were associated with cancer susceptibility for IHC.	2013	[87]
Lung cancer	NBS1	✓ Variants of NBS1 were related with increased risk for lung cancer.	2015	[86]
	NBS1	✓ Functional polymorphisms of NBS1 were related with increased risk for lung cancer.	2014	[90]
Myeloid leukemia	RAD50	✓ Alteration of RAD50 was found in myeloid leukemia.	2019	[81]
Neuroblastoma	MRE11	✓ Alterations in MRE11 was frequent in neuroblastoma.	2017	[80]
NPC	NBS1	✓ Polymorphism of NBS1 is associated with development and status of NPC.	2011	[89]
Ovarian Cancer	MRN	✓ Lack of MRN detection occurred frequently in ovarian cancer.	2017	[76]
	MRE11, RAD50	✓ Pathogenic or likely pathogenic variants were detected in patients.	2018	[75]
	MRE11	✓ Low expression of MRE11 was associated with cancer developments.	2014	[94]
	RAD50	x RAD50 was ruled out as a risk factor.	2017	[91]
Prostate cancer	NBS1	✓ Variants of NBS1 were prevalent in familial prostate cancer.	2018	[85]
Renal cell carcinoma	NBS1	✓ Mutation in NBS1 functional domains was related with susceptibility to renal cell carcinoma.	2015	[88]

✓ Evidence supporting the tumor suppressing role of MRN; x not supporting or opposite conclusions; *HBOC* hereditary breast and ovarian cancer, *IHC* intrahepatic cholangiocarcinoma, *NPC* nasopharyngeal carcinoma

### MRN alterations and cancer chemo/radiotherapy treatment

#### Chemo/radiotherapy

Genomic instability is a hallmark of cancers and renders a great propensity for malignant cells to suffer from genotoxic agents such as DNA-damaging chemotherapeutics and ionizing radiation. Based on this principle, strategies of many chemotherapeutics, such as platinum-based drugs and nucleoside analogs, are used to directly or indirectly interfere with DNA bioprocess and to ultimately lead to DNA damage and cell apoptosis [99, 100]. Despite their broad applications in anticancer treatment, instinct or developed chemo/radiotherapy resistances are common and greatly restrict their applications. Identification of therapy-sensitive populations and combination with other anticancer therapeutics are expected to overcome these problems. Defects in DDR pathways offer the potential for a broader yet more accurate therapeutic avenues in which tailoring treatments according to patients’ genomic peculiarities can be developed.

Alterations of MRN complex component protein have been found to be engaged in various mechanisms of chemo/radio-resistance in multiple tumor cells [101–103].

##### DNA damaging chemotherapy

Cisplatin and gemcitabine are both genotoxic agents that interfere with DNA bioprocess [99, 100]. In bladder cancer cells, downregulation of MRE11 is reportedly responsible for the synergistic effects elicited by combination of cisplatin and gemcitabine [104]. Analysis of gastric cancer specimens of surgical resection after chemotherapy revealed that low expression level of MRN complex is associated with strong responses to chemotherapy and surgical resection [105]. In vitro experiments showed that disruption of MRN complex or its components can confer sensitivity to cisplatin in cancer cells [106, 107] with increase in DNA damage and cell toxicity.

DNA topoisomerase inhibitors exert their anticancer effect by trapping the topoisomerase-DNA covalent complex and converting the enzyme into a cytotoxic covalently-linked protein adduct on DNA, causing the stalled DNA replication and the production of DSBs [108]. As discussed above, MRE11, along with CtIP-BRCA1 complex, plays important roles in processing blocked topoisomerase-DNA ends caused by topoisomerase inhibitors [34, 109]. In addition to removal of adducts from DNA ends catalyzed by MRE11, MRN complex also plays a critical role in activation of cell cycle checkpoint control in response to stalled replication fork [110, 111]. As checkpoint activation provides time for repair of the damage, defects in checkpoint activation in tumor cells may result in sensitivity to therapeutics. With its dominant role in cellular responses to inhibition of replication fork, MRN is found to be required for cellular resistance to etoposide, which exerts its anti-cancer effect by inducing topoisomerase II-DNA adducts [34, 112]. Transfection of MRE11 in MRN-defective cancer cells rescues its deficiency in S-phase arrest in response to topoisomerase I inhibitor [97]. Recently, it has been proved that their ability of nuclease-dependent removal of incorporated adducts from replicating DNA also contributes to cellular tolerance to chain–terminating nucleoside analogs [113].

Decreased levels of MRE11 in combination with elevated chromosome mis-segregation portend a markedly enhanced response to chemoradiation therapy in rectal adenocarcinoma [114].

##### Poly (ADP-ribose) polymerase inhibitor (PARPi)

Synthetic lethal (SL) is a concept describing the situation in which either one or two alterations in genes have little effect on cells or orgasms alone but a combination of these defects is potent enough to induce cell death [115]. SL in BRCA-deficient cells induced by PARPi is probably the most well-known example [9, 116]. BRCA1 and BRCA2 genes are critical components of HR repair for DSB. Defects in HR lead to the shift from HR to the NHEJ pathway, which can ultimately lead to genomic instability and cell death [117]. During tumorigenesis, the wild-type allele of BRCA is lost, while BRCA remains heterozygous in normal tissues, and this opens a selective therapeutic avenue to PARP inhibitors. Because PARP is an important factor of single strand break (SSB) repair, inhibition of PARP activity prevents SSB repair, blocks DNA replication, results in DSB, and confers cytotoxicity, radio- and chemo-sensitivity to cancer cells [116]. The application of PARPi in BRCA-mutant tumor cells is 1,000 times more potent than in BRCA-wild type cells [118]. PARP inhibitors are also tested in cancers lacking BRCA deficiency, but with defects in HR caused by alterations in other genes related to HR. In fact, defects in some HR genes, such as ATM and ATR, have been shown to cause improved sensitivity to PARPi [119].

The relationship between MRN complex and PARPi has also been explored in various studies. In BRCA wild-type (BRCAwt) ovarian cancer patients, 18% of the patients exhibit RAD50 deletion, and this deletion is associated with significantly better overall survival (OS) and progression-free survival (PFS). In ovarian cell lines, deletion of RAD50 copy number leads to better responses to PARP inhibitors. These results suggest that in BRCAwt patients, expression level of RAD50 might be employed as a candidate marker for survival and responses to PARP inhibitors [120]. While loss of MRE11 has been discovered in about 30% of endometrial cancer patients, its depletion also confers sensitivity to PARPi in vitro, indicating that it might serve as a synthetic lethal gene with PARP [121]. It was also reported that defects in the HR pathway (RAD50 defect) is associated with better responses to PARPi in malignant pleural mesothelioma [122]. In neuroblastoma patients, alterations in DDR-associated genes, including MRE11, account for about 50% of the analyzed samples, and this defect may contribute to induction of synthetic lethality with PARP inhibitor, providing an opportunity for PARP inhibitor treatment [80]. In some colorectal tumors, microsatellite instability leads to mutations in MRE11, and this mutational status correlates with PARPi sensitivity [123].

Together, these findings suggest that defects in MRN complex may synergize with PARPi and elevate its efficacy. This speculation is further supported by both *in vitro* and *in vivo* experiments. Inhibitor treatment [124], reduction of expression by siRNA or expression of mutant form [125] of MRN component successfully sensitizes cancer cell lines to PARPi. Lajud *et al* showed that in a mouse model of head and neck squamous cell carcinoma, MRN complex disruption in combination with PARPi leads to accumulation of lethal DSBs, significant shortening of telomere length and, ultimately, regression of BRCA-proficient xenografts [126]. Thus, the potential of MRN complex for SL with PARPi deserves more exploration and might serve as an opportunity for precision medicine similar to the tailoring of PARPi for BRCA-deficient patients.

##### Ionizing radiation

Ionizing radiation causes a spectrum of damages in cells, with DSB being the most lethal type [5]. HR and NHEJ are the two major pathways responsible for DSB repair. Deficiency in either pathway is believed to be related to increased radiosensitivity [9]. ATM and ATR have vital roles in HR and NHEJ pathways. MRN complex is essential for mediating DSB repair and does so by interacting with ATM and ATR in addition to directing the choice of the repair pathway [24].

Overexpression of MRN complex is capable of conferring radioresistance to rectal cancer. High level expression of MRN complex correlates with worse disease-free and overall survival in both postoperative and neoadjuvant radiotherapy group [127]. Targeting RAD50 sensitizes nasopharyngeal carcinoma and colorectal cancer cells to ionizing radiation [128, 129].

Tumor hypoxia has a strong impact on tumor malignancy and treatment resistance. Cowman *et al* [130] found that downregulated expression of NBS1 and MRE11 is involved in hypoxia-induced chemoresistance, inhibition of the DSB pathway and p53 activation in medulloblastoma cells [130]. Kuo *et al* showed that NBS1 is capable of stabilizing hypoxia inducible factor-1a and promoting cancer cell migration and invasion under ionizing radiation [131]. In this perspective, although deficiency in MRN may lead to defects in DDR and increased radiosensitivity, MRN complex may also influence radiosensitivity in an opposite way through other undefined mechanisms.

It has been reported that high levels of MRE11 predict the outcome after radical radiotherapy [132, 133]. However, Walker *et al* later showed that there is no significant association between MRE11 and the rate of success of radiation-based bladder preservation therapy [134]. This inconsistency may be attributed to the lack of an automated quantitative methods or standard reassessment of MRE11.

MRN complex is also linked to treatment-induced toxicity. The pediatric medulloblastoma patients that harbor defects in DNA repair genes, including RAD50 and NBS1, are more vulnerable to treatment-related toxicity [135], suggesting that special vigilance may be needed for patients with MRN defect in chemoradiotherapy.

#### Prognosis

The expression of MRN complex is also linked to the prognosis of cancer. Ho *et al* found that elevated expression of MRN complex in rectal cancer correlates strongly with poor disease-free survival (DFS) and OS [127, 136]. For gastric carcinoma patients, overexpression of MRN is linked to metastasis, chemoresistance and poor OS and PFS [105, 137]. These results suggest that targeting MRN complex in malignant tumors may be an option for treatment.

In pancreatic cancer and uterine serous carcinoma, patients harboring germline variants in DSB repair genes (RAD50, NBS1) tend to have superior survival [138, 139]. In high-grade ovarian cancer, high expression of NBS1 and RAD50 correlates with improved outcome. However, in samples of patients following neoadjuvant chemotherapy, the elevation of HR genes such as BRCA2 is associated with poor outcome [140], suggesting that the choice of molecular markers should vary accordingly. In chemotherapy, higher levels of NBS1 has also been reported to correlate with shorter OS and overall response rate [141]. Polymorphism of NBS1 also correlates with survival in renal cell carcinoma [88]. In melanoma, overexpression of NBS1 has been proved to be linked to metastasis [142].

Low RAD50 expression is associated with poor disease survival and OS in early stage/low-grade rectal cancer patients [143] and colorectal mucinous adenocarcinoma [144]. In non-small cell lung cancer, upregulation of RAD50 may be used to predict shorter RFS [145]. In melanoma patients, RAD50 is the gene most significantly associated with survival among those analyzed, with a higher level of expression linked to poorer outcome [146]. In BRCA1/2-negatice breast cancer patients, although RAD50 pathologic germline mutations poses no increased risk of cancer, it is nevertheless associated with unfavorable survival [147].

It should be noted that expression of the complex may confer different effects under distinct contexts. Conflicting results have been found in studies of gastric cancer with MRE11. Kim *et al* found that low MRE11 expression is associated with worse OS in sporadic gastric cancer [78]. Nevertheless, Li *et al* reported adverse findings [137]. Ihara et al [148] and Pavelitz et al [149] found that MRE11-negatice colorectal cancer patients achieve significantly better tumor reduction compared with the MRE11-positive population. In prostate cancer, high MRE11 expression correlates with progression [150]. It may be that MRE11 expression confers different effects under distinct contexts. Also, the endonuclease and exonuclease function of MRE11 complicates its role in cancer. As discussed earlier, MRE11 might induce chemoresistance by removing covalent adducts from DNA ends. It can also counteract tumor development by degrading stalled replication fork, which is frequently seen in cancer, and inhibition of the degradation of RF may induce chemoresistance [37] [45]. It remains uncertain which function of MRE11 or the MRN complex plays a dominant role in tumor progression.

These conflicting reports on the potential relations between each component of the complex (or the MRN complex as a whole) and the prognosis of different cancer types suggest that there is still no consensus on the role of MRN complex in cancer prognosis. Because prognosis of cancer can be influenced by various factors such as the stage, the treatment received, and so on. A more detailed discussion of the role of MRN complex under specific conditions may better address these roles.

### Targeting MRN complex in cancer

To date, although there is no consensus on the role of MRN complex in tumorigenesis and prognosis, the majority of the results support its potential application as a target in cancer treatment.

#### Genetic modulation

It was reported that targeting MRN complex can halt cancer cell growth and migration [151] and sensitize tumor cells to cisplatin, indicating that genetic modulation of MRN complex might open new avenues for cancers treatment [152]. NBS patients with mutations in NBS1 gene are susceptible to malignancy. Using antisense oligonucleotides against NBS1 gene, Salewsky *et al* [153] enforce alternative splicing of NBS1 gene in NBS patient cells and generate an internally deleted p80-nibrin protein, which skips the mutated exon, carries both amino terminal and carboxyl terminal domains, and possesses undisguisable DNA replication characteristics compared with control NBS1 protein. The same antisense sequences, when injected into humanized NBS1 murine mouse model, lead to efficient alternative splicing, verifying the efficiency of directed exon skipping *in vivo*. These results prove that the use of antisense oligonucleotides can be a potential cancer prophylaxis [153]. Ohara *et al* reported that ectopic expression of FHA-domain mutated NBS1 sensitizes cancer cells to radiation therapy through suppression of HR [154].

siRNA knockdown of RAD50 sensitizes NSCLC cells to radiation therapy in vitro [145]. In breast cancer cells, RAD50 silencing sensitizes cells to DNA damaging agents [155]. By using a dominant negative adenoviral vector containing a mutant RAD50 gene, Abuzeid *et al* showed that downregulation of MRN complex can increase the sensitivity of squamous cell carcinoma cells to platinum-based drugs [107]. Chang *et al* used recombinant adenovirus containing a mutant RAD50 gene to disrupt MRN function and found that this treatment increases radiosensitivity in vitro and decreases tumor growth and accelerates tumor regression *in vivo* [128]. Combination of mutant RAD50 therapy and cisplatin causes dramatic tumor regression in cisplatin-resistant human squamous cell cancer xenografts. These results suggest that RAD50 disruption might be a novel chemosensitizing approach for cancer therapy involving chemoresistance [107].

Over the past two decades, an increasing number of microRNAs (miRNAs) and small noncoding RNAs have been identified as regulators of tumorigenesis and sensitivity to radiation therapy or genotoxic chemotherapies via modulation of DDR [156]. Espinosa-Diez *et al* found that genotoxic stress-induced miR-494 production downregulates MRN complex and DNA repair, resulting in senescence and pathologic angiogenesis disrupt [157].

#### Oncolytic virus

Oncolytic virotherapy employs lytic viruses that replicate selectively in cancer cells. There is a great potential that these viruses can be used along with other anticancer therapeutics. In certain type of oncolytic virotherapy, the ability of various oncolytic adenoviruses to interfere with MRN complex and inhibit the DNA damage response has been proved effective. This has raised great interest in combining this therapy with other DNA damaging treatments - for example, radiotherapy [158].

Stracker *et al* [51] first demonstrated that cells infected with adenovirus serotype 5 (Ad5) lead to the downregulation of intracellular MRN level. This decreased expression is caused by enhanced protein turnover mediated by the Ad5 E4orf6-E1b55K complex. Also, Ad5 F4orf3 and E4orf6 can cause relocation of MRN complex to the cytoplasm and accelerate its degradation and inactivation [52]. Blackford *et al* [159] found that the adenoviral E1B55kDa protein can also exert the function of MRN inhibition in cooperation with E4 protein to create the optimal intracellular environment for viral protein synthesis.

A modified oncolytic adenovirus mutant that lacks an antiapoptotic gene (E1B19K) is found effective in sensitizing pancreatic cells to DNA damage-inducing drugs [160]. Subsequent studies revealed that Telomelysin, a telomerase-dependent oncolytic adenovirus, can produce profound radiosensitizing effects by inhibiting MRN complex via the E1B55kDa protein [161]. Over 30 preclinical studies have reported the antitumor efficacy of Telomelysin against various tumors. A phase I/II study of Telomelysin in combination with radiotherapy for the treatment of esophageal cancer is now ongoing in Japan [162]. The synergetic effect of other oncolytic adenovirus has also been reported elsewhere [163, 164].

#### Modulation of protein translocation

Poruchynsky *et al* showed that proteins trafficking of MRN components along microtubules can be blocked by microtubule-targeting agents [165]. Retaining MRN in the cytoplasm can sensitize cancer cells to genotoxic treatments, mainly by interfering with DDR. These results indicate that inhibition of MRN complex can also be accomplished by interfering with their translocation.

Chen *et al* reported that the ability of the loading of MRN complex onto DSB sites can be inhibited by phosphorylation of MRE11 at S67, which blocks its binding to DSB. Accordingly, mutation at S767 renders MRN complex functional, restores DNA damage-induced cell-cycle arrest and extends the utility of genotoxic treatment [166].

#### Inhibitors

Among several inhibitors of MRN complex, mirin is the most studied. Mirin inhibits MRE11-associated exonuclease activity, prevents MRN-dependent ATM activation, and abolishes the G2/M checkpoint and homology-dependent repair in mammalian cells [167]. Its potential in sensitizing cancer cells towards genotoxic agents have been proved by independent studies. In malignant glioma, mirin sensitizes tumor cells towards lomustine, a DNA-targeting agent, and increases apoptosis and necrosis [168]. In prostate cancer, mirin inhibits androgen-dependent transcription and growth of cancer cells [169]. In MYCN-amplified neuroblastoma cells, higher MRE11 expression predicts poorer prognosis. Upon depletion or inhibition of MRE11 by knockdown or use of mirin, the accumulation of replication stress and DNA damage can be induced and results in significant impairment of tumor growth in vivo, suggesting the potential role of MRE11 inhibition in treating MYCN-amplified neuroblastoma [170]. Mirin also enhances the efficacy of alkylating drugs in glioblastoma cells [168]. Derivatives of mirin have been used for the studies of MRE11 endonuclease and exonuclease [24] , while their potential function in cancer treatment remains poorly explored.

The histone deacetylase (HDAC) inhibitor Panobinostat can downregulate MRE11 and other proteins associated with homologous recombination and radiosensitize tumor cells [102, 171].

Pomegranate extract can inhibit cell proliferation and stimulate apoptosis in breast cancer cells. This anticancer effect may be mediated by its ability to downregulate the expression of molecules of MRN complex and other important genes involved in DSB repair [172].

Heat shock protein 90 (Hsp90) is an evolutionary conserved molecular chaperone for numerous oncoproteins and is considered as an important facilitator of oncogenes. Its inhibitor compromises the ability of the MRN components to form nuclear foci and diminish the interaction between NBS1 and ATM, thus abrogating DSB repair in cancer cells [173]. Targeting Hsp90 may represent a new approach for anti-cancer therapy.

## Conclusions

With its important role in DDR, the function of MRN complex in cancer development and its potential as anticancer target has been widely explored in various types of cancer. It maintains genome stability and prevents malignant transformation in normal cells, but also functions in cancer cells. Many questions remain to be addressed with respect to its role in cancer. For example, there is little information about how MRN complex recognizes DNA ends, and a better delineation of the process should lead to a better understanding of its function. Also, the role of MRN complex in DSB and immune responses needs to be better explored. It is still controversial with respect to its dual role in tumorigenesis and prognosis. It appears that MRN complex is not critical for the development of cancer because most researches tend to consider the MRN complex as risk genes with moderate penetrance. Of note, most of the previous studies have been focused on investigating the relation between a single gene of the complex and cancer risk while often neglecting the state of the entire complex. Because each protein in the complex interacts with one another, the sole focus on individual component might yield information subjected to misinterpretation. To solve this issue, a standard for measuring and evaluate MRN complex as a whole is needed. Furthermore, combination of MRN disruption with PARPi appears to be a promising avenue for precision medicine. This might be realized through implement of oncolytic virotherapy or potent inhibitors. However, the exact synergistic efficacy of MRN complex deficiency and PARPi (or other treatments) require additional evidence at both the basic research and clinical level.

## Data Availability

Not applicable

## Abbreviations

ABC: ATP-binding cassette
Ad5: Adenovirus serotype 5
ATLD: Ataxia-telangiectasia-like disorder
ATM: Ataxia-telangiectasia mutated
ATR: ATM-and-Rad3-related
BRCAwt: BRCA wild type
BRCT: Breast cancer-associated 1 C
DBD: DNA binding domain
DDR: DNA damage response
DFS: Disease-free survival
DSB: Double strand break
FHA: Fork-head associated
HR: Homologous recombination
IHC: Intrahepatic cholangiocarcinoma
MRE11: Meiotic recombination 11 homolog 1
MRN: MRE11-RAD50-NBS1
NBS1: Nijmegen breakage syndrome protein 1
NHEJ: Non-homologous end joining
OR: Odds ratio
OS: Overall survival
PFS: Progression-free survival
RF: Replication fork
SL: Synthetic lethal

## References

[1] Lord CJ, Ashworth A (2012). The DNA damage response and cancer therapy. Nature..

[2] Hoeijmakers JH (2001). Genome maintenance mechanisms for preventing cancer. Nature..

[3] Pearl LH, Schierz AC, Ward SE, Al-Lazikani B, Pearl FM (2015). Therapeutic opportunities within the DNA damage response. Nature Rev Cancer..

[4] Jackson SP, Bartek J (2009). The DNA-damage response in human biology and disease. Nature..

[5] Roos WP, Kaina B (2013). DNA damage-induced cell death: From specific DNA lesions to the DNA damage response and apoptosis. Cancer lett..

[6] Freund A, Orjalo AV, Desprez PY, Campisi J (2010). Inflammatory networks during cellular senescence: causes and consequences. Trends Mol Med..

[7] Roos WP, Thomas AD, Kaina B (2016). DNA damage and the balance between survival and death in cancer biology. Nature Rev Cancer..

[8] Hanahan D, Weinberg RA (2011). Hallmarks of cancer: the next generation. Cell..

[9] O’Connor MJ (2015). Targeting the DNA Damage Response in Cancer. Mol Cell..

[10] Minchom A, Aversa C, Lopez J (2018). Dancing with the DNA damage response: next-generation anti-cancer therapeutic strategies. Ther Adv Med Oncol..

[11] Syed A, Tainer JA (2018). The MRE11-RAD50-NBS1 Complex Conducts the Orchestration of Damage Signaling and Outcomes to Stress in DNA Replication and Repair. Annu Rev Biochem..

[12] Deshpande RA, Lee JH, Paull TT (2017). Rad50 ATPase activity is regulated by DNA ends and requires coordination of both active sites. Nucleic Acids Res..

[13] Hopfner KP, Craig L, Moncalian G, Zinkel RA, Usui T, Owen BA (2002). The Rad50 zinc-hook is a structure joining Mre11 complexes in DNA recombination and repair. Nature..

[14] Lavin MF, Kozlov S, Gatei M, Kijas AW (2015). ATM-Dependent Phosphorylation of All Three Members of the MRN Complex: From Sensor to Adaptor. Biomolecules..

[15] Iijima K, Ohara M, Seki R, Tauchi H (2008). Dancing on Damaged Chromatin: Functions of ATM and the RAD50/MRE11/NBS1 Complex in Cellular Responses to DNA Damage. J Radiat Res..

[16] Blackford AN, Jackson SP (2017). ATM, ATR, and DNA-PK: The Trinity at the Heart of the DNA Damage Response. Mol Cell..

[17] Rotman G, Shiloh Y (1999). ATM: A mediator of multiple responses to genotoxic stress. Oncogene..

[18] Uziel T, Lerenthal Y, Moyal L, Andegeko Y, Mittelman L, Shiloh Y (2003). Requirement of the MRN complex for ATM activation by DNA damage. Embo j..

[19] Oh Julyun, Symington Lorraine (2018). Role of the Mre11 Complex in Preserving Genome Integrity. Genes.

[20] Williams GJ, Lees-Miller SP, Tainer JA (2010). Mre11-Rad50-Nbs1 conformations and the control of sensing, signaling, and effector responses at DNA double-strand breaks. DNA Repair (Amst)..

[21] Williams RS, Moncalian G, Williams JS, Yamada Y, Limbo O, Shin DS (2008). Mre11 Dimers Coordinate DNA End Bridging and Nuclease Processing in Double-Strand-Break Repair. Cell..

[22] Das D, Moiani D, Axelrod HL, Miller MD, McMullan D, Jin KK (2010). Crystal Structure of the First Eubacterial Mre11 Nuclease Reveals Novel Features that May Discriminate Substrates During DNA Repair. J Mol Biol..

[23] Hopfner KP, Karcher A, Craig L, Woo TT, Carney JP, Tainer JA (2001). Structural biochemistry and interaction architecture of the DNA double-strand break repair Mre11 nuclease and Rad50-ATPase. Cell..

[24] Shibata A, Moiani D, Arvai AS, Perry J, Harding SM, Genois MM (2014). DNA double-strand break repair pathway choice is directed by distinct MRE11 nuclease activities. Mol Cell..

[25] Hopfner KP, Karcher A, Shin DS, Craig L, Arthur LM, Carney JP (2000). Structural biology of Rad50 ATPase: ATP-driven conformational control in DNA double-strand break repair and the ABC-ATPase superfamily. Cell..

[26] Park YB, Hohl M, Padjasek M, Jeong E, Jin KS, Krezel A (2017). Eukaryotic Rad50 functions as a rod-shaped dimer. Nat Struct Mol Biol..

[27] Lee JH, Paull TT (2004). Direct activation of the ATM protein kinase by the Mre11/Rad50/Nbs1 complex. Science..

[28] Difilippantonio S, Celeste A, Kruhlak MJ, Lee Y, Difilippantonio MJ, Feigenbaum L (2007). Distinct domains in Nbs1 regulate irradiation-induced checkpoints and apoptosis. J Exp Med..

[29] Liu Y, Sung S, Kim Y, Li F, Gwon G, Jo A (2016). ATP-dependent DNA binding, unwinding, and resection by the Mre11/Rad50 complex. Embo J..

[30] Chen L, Trujillo K, Ramos W, Sung P, Tomkinson AE (2001). Promotion of Dnl4-catalyzed DNA end-joining by the Rad50/Mre11/Xrs2 and Hdf1/Hdf2 complexes. Mol Cell..

[31] Kinner A, Wu W, Staudt C, Iliakis G (2008). Gamma-H2AX in recognition and signaling of DNA double-strand breaks in the context of chromatin. Nucleic Acids Res..

[32] Wang Q, Goldstein M, Alexander P, Wakeman TP, Sun T, Feng J (2014). Rad17 recruits the MRE11-RAD50-NBS1 complex to regulate the cellular response to DNA double-strand breaks. The EMBO J..

[33] Deshpande RA, Lee JH, Arora S, Paull TT (2016). Nbs1 Converts the Human Mre11/Rad50 Nuclease Complex into an Endo/Exonuclease Machine Specific for Protein-DNA Adducts. Mol Cell..

[34] Aparicio T, Baer R, Gottesman M, Gautier J (2016). MRN, CtIP, and BRCA1 mediate repair of topoisomerase II-DNA adducts. J Cell Biol..

[35] Liao S, Tammaro M, Yan H (2016). The structure of ends determines the pathway choice and Mre11 nuclease dependency of DNA double-strand break repair. Nucleic Acids Res..

[36] Lafrance-Vanasse J, Williams GJ, Tainer JA (2015). Envisioning the dynamics and flexibility of Mre11-Rad50-Nbs1 complex to decipher its roles in DNA replication and repair. Prog Biophys Mol Biol..

[37] Zeman MK, Cimprich KA (2014). Causes and consequences of replication stress. Nature Cell Biol..

[38] Oakley GG, Tillison K, Opiyo SA, Glanzer JG, Horn JM, Patrick SM (2009). Physical interaction between replication protein A (RPA) and MRN: involvement of RPA2 phosphorylation and the N-terminus of RPA1. Biochem..

[39] Gatei M, Kijas AW, Biard D, Dork T, Lavin MF (2014). RAD50 phosphorylation promotes ATR downstream signaling and DNA restart following replication stress. Hum Mol Genet..

[40] Aze A, Zhou JC, Costa A, Costanzo V (2013). DNA replication and homologous recombination factors: acting together to maintain genome stability. Chromosoma..

[41] Carr AM, Lambert S (2013). Replication stress-induced genome instability: the dark side of replication maintenance by homologous recombination. J Mol Biol..

[42] Vallerga MB, Mansilla SF, Federico MB, Bertolin AP, Gottifredi V (2015). Rad51 recombinase prevents Mre11 nuclease-dependent degradation and excessive PrimPol-mediated elongation of nascent DNA after UV irradiation. Proc Natl Acad Sci U S A..

[43] Schlacher K, Christ N, Siaud N, Egashira A, Wu H, Jasin M (2011). Double-strand break repair-independent role for BRCA2 in blocking stalled replication fork degradation by MRE11. Cell..

[44] Ying S, Hamdy FC, Helleday T (2012). Mre11-dependent degradation of stalled DNA replication forks is prevented by BRCA2 and PARP1. Cancer Res..

[45] Ray Chaudhuri A, Callen E, Ding X, Gogola E, Duarte AA, Lee JE (2016). Replication fork stability confers chemoresistance in BRCA-deficient cells. Nature..

[46] Marcand S (2014). How do telomeres and NHEJ coexist?. Mol Cell Oncol..

[47] Marcomini I, Gasser SM (2015). Nuclear organization in DNA end processing: Telomeres vs double-strand breaks. DNA Repair (Amst)..

[48] Sabourin M, Zakian VA (2008). ATM-like kinases and regulation of telomerase: lessons from yeast and mammals. Trends Cell Biol..

[49] Shah GA, O'Shea CC (2015). Viral and Cellular Genomes Activate Distinct DNA Damage Responses. Cell..

[50] Kondo T, Kobayashi J, Saitoh T, Maruyama K, Ishii KJ, Barber GN (2013). DNA damage sensor MRE11 recognizes cytosolic double-stranded DNA and induces type I interferon by regulating STING trafficking. Proc Natl Acad Sci U S A..

[51] Stracker TH, Carson CT, Weitzman MD (2002). Adenovirus oncoproteins inactivate the Mre11-Rad50-NBS1 DNA repair complex. Nature..

[52] Liu Y, Shevchenko A, Shevchenko A, Berk AJ (2005). Adenovirus exploits the cellular aggresome response to accelerate inactivation of the MRN complex. J Virol..

[53] Stracker TH, Petrini JHJ (2011). The MRE11 complex: starting from the ends. Nat Rev Mol Cell Biol.

[54] Buis J, Wu Y, Deng Y, Leddon J, Westfield G, Eckersdorff M (2008). Mre11 nuclease activity has essential roles in DNA repair and genomic stability distinct from ATM activation. Cell..

[55] Luo G, Yao MS, Bender CF, Mills M, Bladl AR, Bradley A (1999). Disruption of mRad50 causes embryonic stem cell lethality, abnormal embryonic development, and sensitivity to ionizing radiation. Proc Natl Acad Sci U S A..

[56] Zhu J, Petersen S, Tessarollo L, Nussenzweig A (2001). Targeted disruption of the Nijmegen breakage syndrome gene NBS1 leads to early embryonic lethality in mice. Curr Biol.

[57] van den Bosch M, Bree RT, Lowndes NF (2003). The MRN complex: coordinating and mediating the response to broken chromosomes. EMBO Rep..

[58] Taylor AMR, Groom A, Byrd PJ (2004). Ataxia-telangiectasia-like disorder (ATLD)—its clinical presentation and molecular basis. DNA Repair..

[59] Palmeri S, Rufa A, Pucci B, Santarnecchi E, Malandrini A, Stromillo ML (2013). Clinical course of two Italian siblings with ataxia-telangiectasia-like disorder. Cerebellum (London).

[60] Fiévet Alice, Bellanger Dorine, Valence Stéphanie, Mobuchon Lenha, Afenjar Alexandra, Giuliano Fabienne, Dubois d'Enghien Catherine, Parfait Béatrice, Pedespan Jean‐Michel, Auger Nathalie, Rieunier Guillaume, Collet Agnès, Burglen Lydie, Stoppa‐Lyonnet Dominique, Stern Marc‐Henri (2019). Three new cases of ataxia‐telangiectasia‐like disorder: No impairment of the ATM pathway, but S‐phase checkpoint defect. Human Mutation.

[61] Alsbeih G, Al-Hadyan K, Al-Harbi N (2008). Assessment of carriers’ frequency of a novel MRE11 mutation responsible for the rare ataxia telangiectasia-like disorder. Genet Test..

[62] Digweed M, Reis A, Sperling K (1999). Nijmegen breakage syndrome: consequences of defective DNA double strand break repair. BioEssays.

[63] Digweed M, Sperling K (2004). Nijmegen breakage syndrome: clinical manifestation of defective response to DNA double-strand breaks. DNA Repair (Amst)..

[64] Waltes R, Kalb R, Gatei M, Kijas AW, Stumm M, Sobeck A (2009). Human RAD50 deficiency in a Nijmegen breakage syndrome-like disorder. Am J Hum Genet..

[65] Nicolas E, Bertucci F, Sabatier R, Gonçalves A. Targeting BRCA Deficiency in Breast Cancer: What are the Clinical Evidences and the Next Perspectives? Cancers (Basel). 2018;10(12):506. 10.3390/cancers10120506 PMC6316565 30544963

[66] Bianchini G, Balko JM, Mayer IA, Sanders ME, Gianni L (2016). Triple-negative breast cancer: challenges and opportunities of a heterogeneous disease. Nat Rev Clin Oncol.

[67] Kurian Allison W., Ford James M. (2015). Multigene Panel Testing in Oncology Practice. JAMA Oncology.

[68] Kleibl Z, Kristensen VN (2016). Women at high risk of breast cancer: Molecular characteristics, clinical presentation and management. Breast..

[69] Gupta Gaorav P, Vanness K, Barlas A, Manova-Todorova Katia O, Wen Yong H, Petrini John HJ (2013). The Mre11 Complex Suppresses Oncogene-Driven Breast Tumorigenesis and Metastasis. Mol Cell..

[70] Damiola F, Pertesi M, Oliver J, et al. Rare key functional domain missense substitutions in MRE11A, RAD50, and NBN contribute to breast cancer susceptibility: results from a Breast Cancer Family Registry case-control mutation-screening study. Breast Cancer Res. 2014;16(3):R58. 10.1186/bcr3669 PMC4229874 24894818

[71] Amemiya Y, Bacopulos S, Al-Shawarby M, Al-Tamimi D, Naser W, Ahmed A (2015). A Comparative Analysis of Breast and Ovarian Cancer-related Gene Mutations in Canadian and Saudi Arabian Patients with Breast Cancer. Anticancer Res..

[72] Uzunoglu H, Korak T, Ergul E, Uren N, Sazci A, Utkan NZ (2016). Association of the nibrin gene (NBN) variants with breast cancer. Biomed Rep..

[73] Khan RT, Siddique A, Shahid N, Khokher S, Fatima W (2018). Breast cancer risk associated with genes encoding DNA repair MRN complex: a study from Punjab, Pakistan. Breast Cancer..

[74] Couch FJ, Shimelis H, Hu C, Hart SN, Polley EC, Na J (2017). Associations Between Cancer Predisposition Testing Panel Genes and Breast Cancer. JAMA Oncol..

[75] Koczkowska Magdalena, Krawczynska Natalia, Stukan Maciej, Kuzniacka Alina, Brozek Izabela, Sniadecki Marcin, Debniak Jaroslaw, Wydra Dariusz, Biernat Wojciech, Kozlowski Piotr, Limon Janusz, Wasag Bartosz, Ratajska Magdalena (2018). Spectrum and Prevalence of Pathogenic Variants in Ovarian Cancer Susceptibility Genes in a Group of 333 Patients. Cancers.

[76] Brandt S, Samartzis EP, Zimmermann AK, Fink D, Moch H, Noske A (2017). Lack of MRE11-RAD50-NBS1 (MRN) complex detection occurs frequently in low-grade epithelial ovarian cancer. BMC Cancer..

[77] Zhang H, Liu Y, Zhou K, Zhou C, Zhou R, Cheng C (2016). Genetic variations in the homologous recombination repair pathway genes modify risk of glioma. J Neurooncol..

[78] Kim HS, Kim JW, Hwang IG, Lee HS, Kim WH (2019). Expression of DNA Damage Response Markers in Early-Onset or Familial Gastric Cancers. Asian Pac J Cancer Prev..

[79] Phillips TD, Richardson M, Cheng YS, He L, McDonald TJ, Cizmas LH (2015). Mechanistic relationships between hepatic genotoxicity and carcinogenicity in male B6C3F1 mice treated with polycyclic aromatic hydrocarbon mixtures. Arch Toxicol..

[80] Takagi M, Yoshida M, Nemoto Y, et al. Loss of DNA Damage Response in Neuroblastoma and Utility of a PARP Inhibitor. J Natl Cancer Inst. 2017;109(11):10.1093/jnci/djx062. 10.1093/jnci/djx062 29059438

[81] Simonetti G, Padella A, do Valle IF, Fontana MC, Fonzi E, Bruno S (2019). Aneuploid acute myeloid leukemia exhibits a signature of genomic alterations in the cell cycle and protein degradation machinery. Cancer..

[82] Garcia-Sanz P, Trivino JC, Mota A, Perez Lopez M, Colas E, Rojo-Sebastian A (2017). Chromatin remodelling and DNA repair genes are frequently mutated in endometrioid endometrial carcinoma. Int J Cancer..

[83] Kaymaz Y, Oduor CI, Yu H, Otieno JA, Ong'echa JM, Moormann AM (2017). Comprehensive Transcriptome and Mutational Profiling of Endemic Burkitt Lymphoma Reveals EBV Type–Specific Differences. Mol Cancer Re..

[84] Roset R, Inagaki A, Hohl M, Brenet F, Lafrance-Vanasse J, Lange J (2014). The Rad50 hook domain regulates DNA damage signaling and tumorigenesis. Genes Dev..

[85] Zhen JT, Syed J, Nguyen KA, Leapman MS, Agarwal N, Brierley K (2018). Genetic testing for hereditary prostate cancer: Current status and limitations. Cancer..

[86] KAŁUŻNA EWELINA MARIA, REMBOWSKA JOLANTA, ZIÓŁKOWSKA-SUCHANEK IWONA, ŚWIĄTEK-KOŚCIELNA BOGNA, GABRYEL PIOTR, DYSZKIEWICZ WOJCIECH, NOWAK JERZY STANISŁAW (2015). Heterozygous p.I171V mutation of the NBN gene as a risk factor for lung cancer development. Oncology Letters.

[87] Wang Y, Hong Y, Li M, Long J, Zhao YP, Zhang JX (2013). Mutation inactivation of Nijmegen breakage syndrome gene (NBS1) in hepatocellular carcinoma and intrahepatic cholangiocarcinoma. PLoS One..

[88] Rosinha A, Assis J, Dias F, et al. DNA repair system and renal cell carcinoma prognosis: under the influence of NBS1. Med Oncol. 2015;32(11):255. 10.1007/s12032-015-0701-0 26493193

[89] Zheng J, Zhang C, Jiang L, You Y, Liu Y, Lu J (2011). Functional NBS1 polymorphism is associated with occurrence and advanced disease status of nasopharyngeal carcinoma. Mol Carcinogenesis..

[90] Fang W, Qiu F, Zhang L, Deng J, Zhang H, Yang L (2014). The functional polymorphism of NBS1 p.Glu185Gln is associated with an increased risk of lung cancer in Chinese populations: Case–control and a meta-analysis. Mutat Res Fundam Mol Mech Mutagen.

[91] Lilyquist J, LaDuca H, Polley E, Davis BT, Shimelis H, Hu C (2017). Frequency of mutations in a large series of clinically ascertained ovarian cancer cases tested on multi-gene panels compared to reference controls. Gynecol Oncol..

[92] Aloraifi F, McDevitt T, Martiniano R, McGreevy J, McLaughlin R, Egan CM (2015). Detection of novel germline mutations for breast cancer in non-BRCA1/2 families. The FEBS J.

[93] Castera L, Krieger S, Rousselin A, Legros A, Baumann JJ, Bruet O (2014). Next-generation sequencing for the diagnosis of hereditary breast and ovarian cancer using genomic capture targeting multiple candidate genes. Eur J Hum Genet..

[94] Ye Q, Chen L, Yin X, Liu YJ, Ji Q, Zhao E (2014). Development of serous ovarian cancer is associated with the expression of homologous recombination pathway proteins. Pathol Oncol Res..

[95] Regal JA, Festerling TA, Buis JM, Ferguson DO (2013). Disease-associated MRE11 mutants impact ATM/ATR DNA damage signaling by distinct mechanisms. Hum Mol Genet..

[96] Podralska M, Ziolkowska-Suchanek I, Zurawek M, Dzikiewicz-Krawczyk A, Slomski R, Nowak J (2018). Genetic variants in ATM, H2AFX and MRE11 genes and susceptibility to breast cancer in the polish population. BMC Cancer..

[97] Garner KM, Eastman A. Variations in Mre11/Rad50/Nbs1 status and DNA damage-induced S-phase arrest in the cell lines of the NCI60 panel. BMC Cancer. 2011;11:1–13. 10.1186/1471-2407-11-206 PMC3128005 21619594

[98] Spehalski E, Capper KM, Smith CJ, Morgan MJ, Dinkelmann M, Buis J (2017). MRE11 Promotes Tumorigenesis by Facilitating Resistance to Oncogene-Induced Replication Stress. Cancer Res..

[99] de Sousa CL, Monteiro G (2014). Gemcitabine: metabolism and molecular mechanisms of action, sensitivity and chemoresistance in pancreatic cancer. Eur J Pharmacol..

[100] Dasari S, Bernard TP (2014). Cisplatin in cancer therapy: Molecular mechanisms of action. Eu J Pharmacol.

[101] Khongkow P, Karunarathna U, Khongkow M, Gong C, Gomes AR, Yague E (2014). FOXM1 targets NBS1 to regulate DNA damage-induced senescence and epirubicin resistance. Oncogene..

[102] Nicholson J, Jevons SJ, Groselj B, Ellermann S, Konietzny R, Kerr M (2017). E3 Ligase cIAP2 Mediates Downregulation of MRE11 and Radiosensitization in Response to HDAC Inhibition in Bladder Cancer. Cancer Res..

[103] Li Z, Li J, Kong Y, Yan S, Ahmad N, Liu X (2017). Plk1 Phosphorylation of Mre11 Antagonizes the DNA Damage Response. Cancer Res..

[104] da Silva GN, de Camargo EA, Savio AL, Salvadori DM (2014). MRE11A and SKP2 genes are associated with the increased cytotoxicity induced by the synergistic effects of cisplatin and gemcitabine in bladder cancer cells. Mol Biol Rep..

[105] Altan B, Yokobori T, Ide M, Bai T, Yanoma T, Kimura A (2016). High Expression of MRE11-RAD50-NBS1 Is Associated with Poor Prognosis and Chemoresistance in Gastric Cancer. Anticancer Res..

[106] Araki K, Yamashita T, Reddy N, Wang H, Abuzeid WM, Khan K (2010). Molecular disruption of NBS1 with targeted gene delivery enhances chemosensitisation in head and neck cancer. Br J Cancer..

[107] Abuzeid WM, Jiang X, Shi G, Wang H, Paulson D, Araki K (2009). Molecular disruption of RAD50 sensitizes human tumor cells to cisplatin-based chemotherapy. J Clin Invest..

[108] Delgado JL, Hsieh C-M, Chan N-L, Hiasa H (2018). Topoisomerases as anticancer targets. Biochem J.

[109] Hartsuiker E, Neale MJ, Carr AM (2009). Distinct requirements for the Rad32(Mre11) nuclease and Ctp1(CtIP) in the removal of covalently bound topoisomerase I and II from DNA. Mol Cell..

[110] Lee J, Dunphy WG, Solomon MJ (2013). The Mre11-Rad50-Nbs1 (MRN) complex has a specific role in the activation of Chk1 in response to stalled replication forks. Mol Biol Cell..

[111] Takemura H, Rao VA, Sordet O, Furuta T, Miao Z-H, Meng L (2006). Defective Mre11-dependent Activation of Chk2 by Ataxia Telangiectasia Mutated in Colorectal Carcinoma Cells in Response to Replication-dependent DNA Double Strand Breaks. J Biol Chem.

[112] Hoa NN, Shimizu T, Zhou ZW, Wang ZQ, Deshpande RA, Paull TT (2016). Mre11 Is Essential for the Removal of Lethal Topoisomerase 2 Covalent Cleavage Complexes. Mol Cell..

[113] Mohiuddin M, Rahman MM, Sale JE, Pearson CE (2019). CtIP-BRCA1 complex and MRE11 maintain replication forks in the presence of chain terminating nucleoside analogs. Nucleic Acids Res..

[114] Zaki BI, Suriawinata AA, Eastman AR, Garner KM, Bakhoum SF (2014). Chromosomal instability portends superior response of rectal adenocarcinoma to chemoradiation therapy. Cancer..

[115] Ashworth A, Lord Christopher J, Reis-Filho JS (2011). Genetic Interactions in Cancer Progression and Treatment. Cell..

[116] Lord CJ, Ashworth A (2017). PARP inhibitors: Synthetic lethality in the clinic. Science..

[117] Ceccaldi R, Rondinelli B, D’Andrea AD (2016). Repair Pathway Choices and Consequences at the Double-Strand Break. Trends Cell Biol.

[118] Farmer H, McCabe N, Lord CJ, Tutt AN, Johnson DA, Richardson TB (2005). Targeting the DNA repair defect in BRCA mutant cells as a therapeutic strategy. Nature..

[119] Lord CJ, Ashworth A (2016). BRCAness revisited. Nat Rev Cancer..

[120] Zhang M, Liu G, Xue F, Edwards R, Sood AK, Zhang W (2016). Copy number deletion of RAD50 as predictive marker of BRCAness and PARP inhibitor response in BRCA wild type ovarian cancer. Gynecol Oncol..

[121] Koppensteiner R, Samartzis EP, Noske A, von Teichman A, Dedes I, Gwerder M (2014). Effect of MRE11 loss on PARP-inhibitor sensitivity in endometrial cancer in vitro. PLoS One..

[122] Borchert S, Wessolly M, Schmeller J, Mairinger E, Kollmeier J, Hager T (2019). Gene expression profiling of homologous recombination repair pathway indicates susceptibility for olaparib treatment in malignant pleural mesothelioma in vitro. BMC Cancer..

[123] McPherson LA, Shen Y, Ford JM (2014). Poly (ADP-ribose) polymerase inhibitor LT-626: Sensitivity correlates with MRE11 mutations and synergizes with platinums and irinotecan in colorectal cancer cells. Cancer lett.

[124] El Botty R, Coussy F, Hatem R, Assayag F, Chateau-Joubert S, Servely JL (2018). Inhibition of mTOR downregulates expression of DNA repair proteins and is highly efficient against BRCA2-mutated breast cancer in combination to PARP inhibition. Oncotarget..

[125] Vilar E, Bartnik CM, Stenzel SL, Raskin L, Ahn J, Moreno V (2011). MRE11 deficiency increases sensitivity to poly(ADP-ribose) polymerase inhibition in microsatellite unstable colorectal cancers. Cancer Res..

[126] Lajud SA, Nagda DA, Yamashita T, Zheng J, Tanaka N, Abuzeid WM (2014). Dual disruption of DNA repair and telomere maintenance for the treatment of head and neck cancer. Clin Cancer Res..

[127] Ho V, Chung L, Singh A, Lea V, Abubakar A, Lim SH (2018). Overexpression of the MRE11-RAD50-NBS1 (MRN) complex in rectal cancer correlates with poor response to neoadjuvant radiotherapy and prognosis. BMC Cancer..

[128] Chang L, Huang J, Wang K, Li J, Yan R, Zhu L (2016). Targeting Rad50 sensitizes human nasopharyngeal carcinoma cells to radiotherapy. BMC Cancer..

[129] Chen C, Wang Y, Mei JF, Li SS, Xu HX, Xiong HP (2018). Targeting RAD50 increases sensitivity to radiotherapy in colorectal cancer cells. Neoplasma..

[130] Cowman S, Fan YN, Pizer B, See V (2019). Decrease of Nibrin expression in chronic hypoxia is associated with hypoxia-induced chemoresistance in some brain tumour cells. BMC Cancer..

[131] Kuo Y-C, Wu H-T, Hung J-J, Chou T-Y, Teng S-C, Wu K-J (2015). Nijmegen breakage syndrome protein 1 (NBS1) modulates hypoxia inducible factor-1α (HIF-1α) stability and promotes in vitro migration and invasion under ionizing radiation. Int J Biochem Cell Biol.

[132] Teo MT, Dyrskjot L, Nsengimana J, Buchwald C, Snowden H, Morgan J (2014). Next-generation sequencing identifies germline MRE11A variants as markers of radiotherapy outcomes in muscle-invasive bladder cancer. Ann Oncol..

[133] Choudhury A, Nelson LD, Teo MT, Chilka S, Bhattarai S, Johnston CF (2010). MRE11 expression is predictive of cause-specific survival following radical radiotherapy for muscle-invasive bladder cancer. Cancer Res..

[134] Walker AK, Karaszi K, Valentine H, Strauss VY, Choudhury A, McGill S (2019). MRE11 as a Predictive Biomarker of Outcome After Radiation Therapy in Bladder Cancer. Int J Radiat Oncol Biol Phys..

[135] Trubicka J, Zemojtel T, Hecht J, Falana K, Piekutowska-Abramczuk D, Ploski R (2017). The germline variants in DNA repair genes in pediatric medulloblastoma: a challenge for current therapeutic strategies. BMC Cancer..

[136] Ho V, Chung L, Revoltar M, Lim SH, Tut TG, Abubakar A (2016). MRE11 and ATM Expression Levels Predict Rectal Cancer Survival and Their Association with Radiotherapy Response. PLoS One..

[137] Li J, Su T, Yang L, Zhang C, He Y. High expression of MRE11 correlates with poor prognosis in gastric carcinoma. Diagn Pathol. 2019;14(1):60. 10.1186/s13000-019-0844-y PMC6587374 31221177

[138] Frimer M, Levano KS, Rodriguez-Gabin A, Wang Y, Goldberg GL, Horwitz SB (2016). Germline mutations of the DNA repair pathways in uterine serous carcinoma. Gynecol Oncol..

[139] Yurgelun MB, Chittenden AB, Morales-Oyarvide V, Rubinson DA, Dunne RF, Kozak MM (2019). Germline cancer susceptibility gene variants, somatic second hits, and survival outcomes in patients with resected pancreatic cancer. Genet Med.

[140] Kessous R, Octeau D, Klein K, Tonin PN, Greenwood CMT, Pelmus M (2018). Distinct homologous recombination gene expression profiles after neoadjuvant chemotherapy associated with clinical outcome in patients with ovarian cancer. Gynecologic Oncol..

[141] Monk BJ, Kaye SB, Poveda A, Herzog TJ, Aracil M, Nieto A (2014). Nibrin is a marker of clinical outcome in patients with advanced serous ovarian cancer treated in the phase III OVA-301 trial. Gynecologic Oncol.

[142] Quan L, Shi J, Tian Y, Zhang Q, Zhang Y, Zhang Y (2015). Identification of potential therapeutic targets for melanoma using gene expression analysis. Neoplasma..

[143] Ho Vincent, Chung Liping, Singh Amandeep, Lea Vivienne, Revoltar Maxine, Lim Stephanie, Tut Thein-Ga, Ng Weng, Lee Mark, de Souza Paul, Shin Joo-Shik, Soon Lee Cheok (2017). Early Postoperative Low Expression of RAD50 in Rectal Cancer Patients Associates with Disease-Free Survival. Cancers.

[144] Wang Mo-Jin, Ping Jie, Li Yuan, Holmqvist Annica, Adell Gunnar, Arbman Gunnar, Zhang Hong, Zhou Zong-Guang, Sun Xiao-Feng (2015). Prognostic Significance and Molecular Features of Colorectal Mucinous Adenocarcinomas. Medicine.

[145] Wang Y, Gudikote J, Giri U, Yan J, Deng W, Ye R (2018). RAD50 Expression Is Associated with Poor Clinical Outcomes after Radiotherapy for Resected Non-small Cell Lung Cancer. Clin Cancer Res..

[146] Gorlov I, Orlow I, Ringelberg C, Hernando E, Ernstoff MS, Cheng C (2018). Identification of gene expression levels in primary melanoma associated with clinically meaningful characteristics. Melanoma Res..

[147] Fan C, Zhang J, Ouyang T, Li J, Wang T, Fan Z (2018). RAD50 germline mutations are associated with poor survival in BRCA1/2-negative breast cancer patients. Int J Cancer..

[148] Ihara K, Yamaguchi S, Ueno N, Tani Y, Shida Y, Ogata H (2016). Expression of DNA double-strand break repair proteins predicts the response and prognosis of colorectal cancer patients undergoing oxaliplatin-based chemotherapy. Oncol Rep..

[149] Pavelitz Thomas, Renfro Lindsay, Foster Nathan R., Caracol Amber, Welsch Piri, Lao Victoria Valinluck, Grady William B., Niedzwiecki Donna, Saltz Leonard B., Bertagnolli Monica M., Goldberg Richard M., Rabinovitch Peter S., Emond Mary, Monnat Raymond J., Maizels Nancy (2014). MRE11-Deficiency Associated with Improved Long-Term Disease Free Survival and Overall Survival in a Subset of Stage III Colon Cancer Patients in Randomized CALGB 89803 Trial. PLoS ONE.

[150] Wang J, Xu WH, Wei Y, Zhu Y, Qin XJ, Zhang HL (2019). Elevated MRE11 expression associated with progression and poor outcome in prostate cancer. J Cancer..

[151] Gao R, Singh R, Kaul Z, Kaul SC, Wadhwa R (2015). Targeting of DNA Damage Signaling Pathway Induced Senescence and Reduced Migration of Cancer cells. J Gerontol A Biol Sci Med Sci..

[152] Li D, Ye L, Lei Y, Wan J, Chen H (2019). Downregulation of FoxM1 sensitizes nasopharyngeal carcinoma cells to cisplatin via inhibition of MRN-ATM-mediated DNA repair. BMB Rep.

[153] Salewsky B, Hildebrand G, Rothe S, Parplys AC, Radszewski J, Kieslich M (2016). Directed Alternative Splicing in Nijmegen Breakage Syndrome: Proof of Principle Concerning Its Therapeutical Application. Mol Ther.

[154] Ohara M, Funyu Y, Ebara S, Sakamoto Y, Seki R, Iijima K (2014). Mutations in the FHA-domain of ectopically expressed NBS1 lead to radiosensitization and to no increase in somatic mutation rates via a partial suppression of homologous recombination. J Radiat Res..

[155] Flores-Pérez A, Rafaelli LE, Ramírez-Torres N, Aréchaga-Ocampo E, Frías S, Sánchez S (2014). RAD50 targeting impairs DNA damage response and sensitizes human breast cancer cells to cisplatin therapy. Cancer Biol Ther..

[156] He M, Zhou W, Li C, Guo M. MicroRNAs, DNA Damage Response, and Cancer Treatment. Int J Mol Sci. 2016;17(12):2087. 10.3390/ijms17122087 PMC5187887 27973455

[157] Espinosa-Diez C, Wilson R, Chatterjee N, Hudson C, Ruhl R, Hipfinger C (2018). MicroRNA regulation of the MRN complex impacts DNA damage, cellular senescence, and angiogenic signaling. Cell Death Dis..

[158] O'Cathail SM, Pokrovska TD, Maughan TS, Fisher KD, Seymour LW, Hawkins MA (2017). Combining Oncolytic Adenovirus with Radiation-A Paradigm for the Future of Radiosensitization. Front Oncol..

[159] Blackford AN, Grand RJA (2009). Adenovirus E1B 55-Kilodalton Protein: Multiple Roles in Viral Infection and Cell Transformation. J Virol.

[160] Pantelidou C, Cherubini G, Lemoine NR, Hallden G (2016). The E1B19K-deleted oncolytic adenovirus mutant AdDelta19K sensitizes pancreatic cancer cells to drug-induced DNA-damage by down-regulating Claspin and Mre11. Oncotarget..

[161] Kuroda S, Fujiwara T, Shirakawa Y, Yamasaki Y, Yano S, Uno F (2010). Telomerase-dependent oncolytic adenovirus sensitizes human cancer cells to ionizing radiation via inhibition of DNA repair machinery. Cancer Res..

[162] Taguchi Satoru, Fukuhara Hiroshi, Todo Tomoki (2018). Oncolytic virus therapy in Japan: progress in clinical trials and future perspectives. Japanese Journal of Clinical Oncology.

[163] Zhang H, Wang F, Mao C, Zhang Z, Fu S, Lu J (2017). Effect of combined treatment of radiation and tissue-specific recombinant oncolytic adenovirus on bladder cancer cells. Int J Radiat Biol..

[164] Rajecki M, af Hallstrom T, Hakkarainen T, Nokisalmi P, Hautaniemi S, Nieminen AI (2009). Mre11 inhibition by oncolytic adenovirus associates with autophagy and underlies synergy with ionizing radiation. Int J Cancer..

[165] Poruchynsky MS, Komlodi-Pasztor E, Trostel S, Wilkerson J, Regairaz M, Pommier Y (2015). Microtubule-targeting agents augment the toxicity of DNA-damaging agents by disrupting intracellular trafficking of DNA repair proteins. Proc Natl Acad Sci U S A..

[166] Chen C, Zhang L, Huang NJ, Huang B, Kornbluth S (2013). Suppression of DNA-damage checkpoint signaling by Rsk-mediated phosphorylation of Mre11. Proc Natl Acad Sci U S A..

[167] Dupré A, Boyer-Chatenet L, Sattler RM, Modi AP, Lee J-H, Nicolette ML (2008). A forward chemical genetic screen reveals an inhibitor of the Mre11–Rad50–Nbs1 complex. Nature Chemical Biol.

[168] Berte N, Piee-Staffa A, Piecha N, Wang M, Borgmann K, Kaina B (2016). Targeting Homologous Recombination by Pharmacological Inhibitors Enhances the Killing Response of Glioblastoma Cells Treated with Alkylating Drugs. Mol Cancer Ther..

[169] Jividen K, Kedzierska KZ, Yang CS, Szlachta K, Ratan A, Paschal BM (2018). Genomic analysis of DNA repair genes and androgen signaling in prostate cancer. BMC Cancer..

[170] Petroni M, Sardina F, Infante P, Bartolazzi A, Locatelli E, Fabretti F (2018). MRE11 inhibition highlights a replication stress-dependent vulnerability of MYCN-driven tumors. Cell Death Dis..

[171] Groselj B, Ruan JL, Scott H, Gorrill J, Nicholson J, Kelly J (2018). Radiosensitization In Vivo by Histone Deacetylase Inhibition with No Increase in Early Normal Tissue Radiation Toxicity. Mol Cancer Ther..

[172] Shirode AB, Kovvuru P, Chittur SV, Henning SM, Heber D, Reliene R (2014). Antiproliferative effects of pomegranate extract in MCF-7 breast cancer cells are associated with reduced DNA repair gene expression and induction of double strand breaks. Mol Carcinog..

[173] Pennisi R, Ascenzi P, di Masi A (2015). Hsp90: A New Player in DNA Repair?. Biomolecules..

